# Exploring the Interplay Between Hypertension and Osteoporosis: A Narrative Review

**DOI:** 10.7759/cureus.97459

**Published:** 2025-11-21

**Authors:** Yolanda Gutierrez, Ronald A Shaju, Alyssa Sepulveda, Seval Coban

**Affiliations:** 1 School of Medicine, University of Texas Rio Grande Valley School of Medicine, Edinburg, USA

**Keywords:** anti-hypertensives, chronic disease managment, hypertension, osteoporosis, pharmacology

## Abstract

Hypertension and osteoporosis are highly prevalent chronic conditions that have significant implications for global health, especially in aging populations. While these conditions have traditionally been viewed as separate, emerging research suggests a strong pathophysiological link between the two. Understanding these shared mechanisms may help improve screening and guide integrated management strategies. This narrative review was conducted between January 2024 and July 2025 using PubMed, ScienceDirect, and Google Scholar. The search strategy used the Boolean string: (“hypertension” OR “high blood pressure”) AND (“osteoporosis” OR “bone loss”), supplemented with pharmacologic terms (“ACE inhibitors,” “angiotensin receptor blockers,” “thiazide diuretics,” “beta-blockers”, “SERMS”, “Bisphosphonates”, “Denosumab”, “romosozumab”, and “teriparatide”). Filters included English language, peer-reviewed human and animal studies when mechanically relevant. A total of 336 articles were retrieved, 143 titles and abstracts were screened, and 61 articles were selected. Data were synthesized qualitatively and organized thematically. Evidence demonstrates that oxidative stress and the renin-angiotensin-aldosterone system (RAAS) activation serve as strong mechanisms linking osteoporosis and hypertension. These pathways promote endothelial dysfunction, osteoclastogenesis, and impaired osteoblast activity. Hormonal disturbances such as estrogen deficiency can further exacerbate both vascular and skeletal deterioration. Several antihypertensive medications, including thiazide diuretics, angiotensin-converting enzyme inhibitors (ACEis), and angiotensin II receptor blockers (ARBs), show bone protective effects; however, most osteoporosis medications exhibit neutral or mixed influence on the cardiovascular system. Hypertension and osteoporosis share connected biological pathways that contribute to both vascular dysfunction and bone loss. Recognizing this connection can encourage therapeutic approaches and address cardiovascular and skeletal health. Further research is needed to define appropriate dual interventions.

## Introduction and background

Hypertension and osteoporosis are two of the most prevalent chronic diseases worldwide, each contributing substantially to morbidity and mortality in aging populations. Although both conditions are well studied independently, their coexistence and potential biological interconnection remain underrecognized. Understanding this relationship is important, as evidence indicates that shared mechanisms may link cardiovascular health and bone metabolism. Hypertension is estimated to contribute to approximately 7.5 million deaths annually worldwide, accounting for nearly 13% of all deaths [1]. Primary hypertension results from a combination of genetic, behavioral, and environmental factors, whereas secondary hypertension arises from underlying conditions such as chronic kidney disease, endocrine disorders, or vascular abnormalities [2].

Osteoporosis is a systemic skeletal disorder characterized by reduced bone mass and architectural degradation, leading to increased fracture risk [3]. Beyond low bone density, recent studies highlight the role of oxidative stress and pro-inflammatory cytokines in disrupting osteoblast/osteoclast balance, linking bone loss with metabolic and vascular processes [4]. Traditional risk factors include older age, female sex, low body weight, inadequate calcium and vitamin D intake, physical inactivity, smoking, and excessive alcohol use [5-7], while chronic inflammation, oxidative stress, and reduced antioxidant defenses have been identified as additional contributors [8]. Lifestyle interventions such as weight-bearing exercise and adequate nutritional intake, along with pharmacologic therapies including bisphosphonates, parathyroid hormone (PTH) analogs, and RANK-L or sclerostin inhibitors, reduce fracture risk [9]. Growing evidence reinforces a connection between hypertension and osteoporosis. In a prospective study of 3,676 untreated women with varying blood pressure levels, higher baseline blood pressure was associated with accelerated bone loss at the femoral neck [10]. Despite continued emerging evidence, there continues to be a limited understanding of the coexistence of these diseases. For instance, some recent studies note that findings are often contradictory, with some studies suggesting hypertension is associated with lower bone mineral density while others report neutral or positive associations [11,12]. Greater recognition of this overlap could promote earlier diagnosis, integrated risk assessment, and more comprehensive management of both conditions. 

## Review

Methods

This narrative review aimed to synthesize current evidence on the shared mechanisms and clinical associations between hypertension and osteoporosis. This review was conducted from January 2024 to July 2025 using PubMed, ScienceDirect, and Google Scholar.

The search strategy used the following combination of terms: (“hypertension” OR “high blood pressure”) AND (“osteoporosis” OR“bone loss”), supplemented with pharmacologic terms including “ACE inhibitors”, “Angiotensin receptor blockers”, “Thiazide diuretics”, “Beta Blockers”, “SERMS”, “Bisphosphonates”, “Denosumab”, “romosozumab”, and “teriparatide”.

Filters were applied to include human and animal studies when mechanistically relevant, peer-reviewed articles, and English-language publications. Reference lists of key articles and reviews were also manually screened to identify eligible studies.

Inclusion criteria were studies that examined pathophysiological mechanisms linking hypertension and osteoporosis, evaluation of antihypertensive medications with the skeletal system, osteoporosis medications with cardiovascular system effects, and lifestyle or hormonal factors affecting both the skeletal and cardiovascular systems.

Exclusion criteria included case reports, small case series with fewer than 20 subjects, non-peer-reviewed articles, studies unrelated to mechanisms or management, and non-English publications.

A total of 336 articles were retrieved. After removal of duplicates (n=37), 143 titles and abstracts were screened, and 61 articles were ultimately included for addressing mechanistic pathways, pharmacologic effects, and clinically relevant associations between osteoporosis and hypertension. Reviewer disagreements were resolved through discussion and consensus.

No formal risk-of-bias tools or quantitative synthesis were used, as this review was a narrative design. No statistical synthesis or meta-analysis was performed due to the narrative nature of the review and the heterogeneity of the included studies. Data was extracted qualitatively and organized into sections, including oxidative stress, renin-angiotensin-aldosterone system (RAAS) activation, hormonal imbalance, lifestyle impacts, and pharmacologic interactions.

Results

Oxidative Stress in Hypertension and Osteoporosis

Oxidative stress is a key pathological factor in both hypertension and osteoporosis. It occurs when the accumulation of reactive oxygen species (ROS) leads to cellular damage [13-15]. Numerous studies identify oxidative stress as a unifying mechanism contributing to vascular remodeling, endothelial dysfunction, and organ damage in hypertension, while also impairing bone formation by stimulating osteoclast activity and suppressing osteoblast differentiation, particularly in aging and estrogen-deficient states [13-15]. Although much of the literature examines these effects separately, several reviews emphasize the mechanistic overlap and propose that targeting redox imbalance may represent a shared therapeutic strategy for both conditions [13-15].

In bone metabolism, oxidative stress promotes osteoclastogenesis and impairs osteoblast function. One key pathway involves NF-κB, a transcription factor activated by inflammatory cytokines and ROS [16-18]. In osteoblasts, NF-κB activation induces the expression of receptor activator of nuclear factor κB ligand (RANKL), which binds to its receptor RANK on osteoclast precursors [16]. This interaction promotes osteoclast differentiation and activation, leading to enhanced bone resorption and contributing to osteoporosis pathogenesis [16]. Studies also suggest that ROS-mediated bone resorption involves activation of mitogen-activated protein kinase (MAPK) pathways, specifically ERK, JNK, and P28, which regulate osteoclast differentiation and survival [19,20]. In parallel, oxidative stress can activate the NLRP3 inflammasome, leading to increased secretion of pro-inflammatory cytokines such as IL1B and IL18; these mediators further amplify osteoclast activity and bone resorption, particularly under inflammatory and diabetic conditions [19,20].

**Figure 1:**
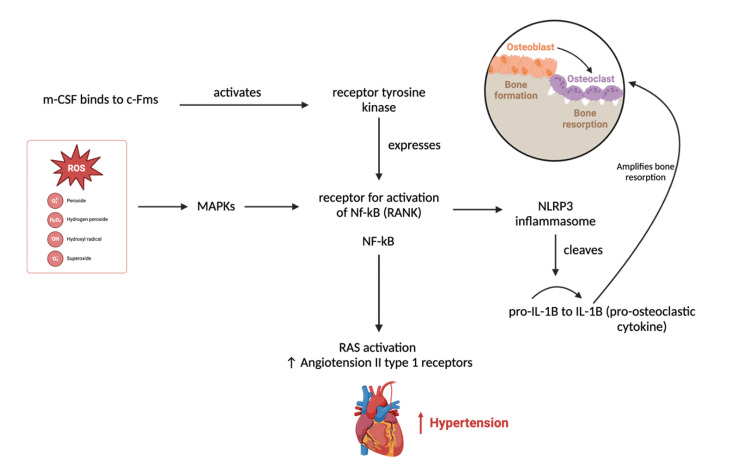
A schematic depicting the dual effect of NF-κB and reactive oxygen species (ROS) on bone resorption and hypertension. Created in BioRender. Sepulveda, A. (2025) https://BioRender.com/g80q939

In hypertension, ROS contribute to endothelial dysfunction, vascular inflammation, and remodeling [21]. ROS reduces nitric oxide bioavailability and promotes smooth proliferation, contributing to the development of hypertension [22]. Similar to osteoporosis, redox-sensitive pathways such as NF-κB and MAPK are upregulated, exacerbating inflammation and vascular injury as observed in Figure 1 [23]. Identical to osteoporosis, redox-sensitive pathways such as NF-κB and MAPK are upregulated, exacerbating inflammation and vascular injury. For example, in hypertensive rodent models, increased ROS in the hypothalamic and paraventricular nucleus activated the NF-κB/AT1R axis, promoting sympathetic tone and RAAS activation [23]. NLRP3 inflammasome activation also plays a role in renal inflammation and hypertension, and its inhibition has been shown to lower blood pressure and renal fibrosis [24]. Similar patterns are observed in humans, where hypertensive patients show elevated NADPH oxidase activity, superoxide production, and NFKB activation in cardiovascular regulatory brain regions, correlating with increased sympathetic outflow [13]. The NLRP3 inflammasome has been documented in both experimental and clinical hypertension, with higher circulating IL-1β B and caspase 1 levels leading to blood pressure elevation [25]. Overall, oxidative stress is a shared mechanism linking hypertension and osteoporosis, with implications for future therapies targeting redox-sensitive pathways.

RAAS in Hypertension and Osteoporosis

The RAAS plays a critical role in regulating blood pressure, fluid balance, and vascular tone and has also been implicated in bone remodeling. RAAS activation begins with renin release from juxtaglomerular cells in the kidney, typically in response to decreased renal perfusion, sympathetic nervous stimulation, or reduced sodium delivery to the distal tubule. Renin catalyzes the conversion of angiotensinogen to angiotensin I, which is subsequently converted to angiotensin II (Ang II) by angiotensin-converting enzyme (ACE), primarily in the lungs [26]. Angiotensin II increases blood pressure primarily by promoting vasoconstriction, sodium retention, and aldosterone secretion; beyond these systemic effects, Ang II also contributes to oxidative stress and NFKB activation, pathways that impair osteoblast differentiation and enhance osteoclastogenesis, thereby linking RAAS activation to both vascular remodeling and bone loss [27-29]. Beyond its systemic effects, RAAS also influences bone metabolism through local tissue-specific activity [30]. Ang II inhibits osteoblast differentiation and promotes osteoclastogenesis, in part via activation of the RANKL pathway and NF-κB signaling [31,32]. Ang II also suppresses the expression of Cbfa1/Runx2, a key transcription factor for osteoblast differentiation, and promotes bone marrow lipid accumulation, which may impair osteogenesis [18,33].

The ACE2/angiotensin-(1-7)/Mas receptor is a counterregulatory arm of the renin-angiotensin system with protective effects on bone [34,35]. In animal models, activation of this pathway promotes osteoblast differentiation, inhibits osteoclastogenesis, and improves bone architecture; these effects are abolished by the Mas receptor antagonist [34,35]. ACE2 and Mas receptor expression have also been detected in human bone and gingival tissues, though direct clinical evidence linking this axis to osteoporosis remains limited. Taken together, these findings highlight the dual role of RAAS in cardiovascular and skeletal systems. Understanding how Ang II and its downstream pathways affect both blood pressure and bone homeostasis opens potential avenues for dual-purpose pharmacologic interventions.

Hormonal Imbalance

Hormonal processes are also implicated in the relationship between osteoporosis and hypertension, especially estrogen and parathyroid hormone [36]. Estrogen has been investigated in randomized controlled trials, such as the Women's Health Initiative, which demonstrated how estrogen replacement in postmenopausal women increases bone mineral density and decreases the risk of fractures [37]. Along with this, they were also able to demonstrate how estrogen deficiency is associated not only with increased bone resorption but also with vascular dysfunction leading to hypertension [37]. Parathyroid analogs such as teriparatide and abaloparatide have also been investigated, demonstrating their use increases bone formation, and chronic elevation of PTH can be associated with bone loss and increased blood pressure due to increased vascular smooth muscle calcium influx [36,38]. Together, these hormonal pathways can highlight the complex interplay between vascular and skeletal health and can both help mitigate the risk of osteoporosis and hypertension.

Lifestyle Impacts

Lifestyle modifications are essential in the prevention and management of both hypertension and osteoporosis, especially as populations age. Although these conditions have different etiologies, they share many modifiable risk factors that can influence both the cardiovascular and skeletal systems. In hypertension, lifestyle interventions such as sodium restriction, decrease in weight, and physical exercise can improve endothelial function, decrease oxidative stress, and decrease blood pressure [39,40]. Along with this, weight-bearing exercise, intake of calcium and vitamin D, and a diet rich in fruits and vegetables can benefit bone formation and decrease bone resorption [39,40]. On the other hand, smoking, heavy alcohol use, and reduced calcium intake can lead to a higher occurrence of fractures, especially in women, as observed in Table 1 [41]. A randomized clinical trial investigating the Dietary Approaches to Stop Hypertension (DASH) diet demonstrated that decreased dietary acid load, such as in foods like meat, cheese, and eggs, can decrease blood pressure in postmenopausal women, along with reducing bone turnover markers such as C-terminal telopeptide (CTX) and procollagen type 1 N-terminal propeptide (P1NP) [42,43]. These findings suggest that dietary patterns provide an ideal area for impacting both diseases. In sum, the combination of lifestyle modifications and medication therapy can provide augmentative effects, providing a low-cost and low-risk approach to the development of both osteoporosis and hypertension. 

**Table 1:** Shared risk factors for hypertension and osteoporosis

Risk Factor	Influence on Hypertension	Influence on Osteoporosis	Preventative Measures
Menopause	Decreased estrogen increases vasoconstriction and reactive oxygen species	Decreased estrogen increases bone resorption and decreases bone formation	Adequate dietary measures and resistance training
Substance abuse (alcohol and smoking)	Increased reactive oxygen species and atherosclerosis	Increased reactive oxygen species	Substance use modulation or cessation
High-fat diet	Atherosclerosis causes vessel damage	Low calcium and vitamin D lead to decreased bone mineralization	Adoption of the Dietary Approaches to Stop Hypertension (DASH) diet
Decreased physical activity	Poor vasodilation and increased arterial stiffness	Reduced bone mineral density	Adoption of weight-bearing exercise

Drugs, Molecular Mechanisms, and Interplay

Pharmacologic treatment plays a role in managing both hypertension and osteoporosis, with several drug classes influencing both vascular and skeletal pathways. Some antihypertensive medications can influence bone metabolism through calcium processing, decreased oxidative stress, and inhibition of the RAAS pathway [44-46]. Conversely, osteoporosis medications can influence cardiovascular physiology through effects on endothelial function, calcium balance, or inflammatory pathways [44-46]. These shared mechanisms provide the biological basis for potential cross-effects between drug classes used for each condition.

Although there is some research about antihypertensive management correlating with bone, medications for osteoporosis involving hypertension are limited. This section examines the major antihypertensive and osteoporosis medications, further expanding on their mechanisms in the cardiovascular and skeletal systems

Thiazide Diuretics

Thiazide diuretics are a first-line treatment for hypertension, and have been associated with reduced fracture risk; however, this is particularly seen in postmenopausal women, with limited data in men [47,48]. They also may have effects on osteoblast differentiation and reduce bone remodeling by decreasing parathyroid hormone levels [49,50]. Although these mechanisms may suggest a benefit in bone, most of the evidence available is from observational or cohort studies demonstrating a positive association rather than a therapeutic benefit. Further clinical research in humans is needed to provide clarification of the specific mechanisms and long-term effects of thiazide diuretics on bone in different populations.

Calcium Channel Blockers (CCBs)

CCBs, particularly dihydropyridines, are widely used for hypertension management. Some CCBs may also exert bone-protective effects, such as cilnidipine, which was found to increase bone density by decreasing osteoclasts [51,52]. However, with other CCBs such as nifedipine, there are mixed results. For example, in vitro studies demonstrate that it can increase alkaline phosphatase activity in osteoblasts, but it would not increase mineral deposition, therefore demonstrating no anabolic effect [53]. However, other animal studies have demonstrated that it can decrease bone density and bone volume [54]. Overall, the effects of CCBs on bone appear to vary by drug class. Almost all the evidence available appears to come from animal models; prospective and randomized controlled human trials are needed to determine a true role in bone metabolism.

ACE Inhibitors (ACEIs)

ACEIs inhibit the conversion of angiotensin I to angiotensin II, thereby preventing vasoconstriction and aldosterone secretion. Some studies suggest that ACEIs may have an effect on protecting bone. For example, in one study, ACEI use was accompanied by higher bone mineral density at the femoral neck and lumbar spine [55]. Among the ACEis available, captopril has demonstrated reduced bone loss in animal studies; it increased bone metabolic markers, increased mineralization, and increased trabecular and cortical bone strength via the ACE-2/Ang1-7/Mas receptor pathway, along with decreased osteoclastogenesis [56-58]. Another ACEi called imidapril has also demonstrated decreased bone loss and osteoclast activation in rats, demonstrating a beneficial effect on osteoporosis [52]. Although these ACEis have demonstrated positive associations, a recent human cohort study has shown they were not associated with improved bone mineral density [59]. Human studies have demonstrated mixed results on lisinopril, enalapril, ramipril, and captopril; some have shown a small reduction in fracture risk; however, others have not shown any association. If there are benefits observed, they are less than angiotensin II receptor blockers (ARBs) [60-63]. Therefore, animal studies might not necessarily reflect the same results as human studies. Although this evidence demonstrates promising results, further research in human studies is necessary to determine the actual benefit of these medications regarding bone loss.

ARBs

ARBs inhibit angiotensin II from binding to its receptor, reducing RAAS-mediated vasoconstriction and sodium retention; a few preclinical studies have demonstrated that ARBs can also reduce osteoclastogenesis and promote osteoblast activity via cAMP-mediated signaling [64,65]. However, this pathway has not been conclusively validated in human studies. Experimental data in rats suggests telmisartan and olmesartan improve bone architecture and increase bone mineral density [66-68]. A recent human cohort study demonstrated that losartan, valsartan, irbesartan, candesartan, and telmisartan are linked to reduced fracture risk and improved bone mineral density in the femoral neck, hip, and lumbar spine, along with decreased osteoclast activity [59]. Studies have also demonstrated a reduced risk of hip fractures in patients taking ARBs [60,69,70]. While ARBs are effective in controlling hypertension and show promise for their capabilities in osteoporosis patients, it would be beneficial for the relationship between osteoporosis and hypertension to be further studied.

Potassium-Sparing Diuretics

Potassium-sparing diuretics, including aldosterone antagonists such as spironolactone and eplerenone, prevent sodium reabsorption and potassium excretion in the distal nephron. These agents may influence bone health, but it's less robust than with thiazides or RAAS blockers. In primary aldosteronism, a cause of osteoporosis, spironolactone slows down bone loss. It increases bone markers such as PINP, bone-specific alkaline phosphatase (BALP), osteocalcin, and tartrate-resistant acid phosphatase (TRAPC) [71]. Spironolactone's anti-mineralocorticoid effect increases serum calcium levels and similarly decreases PTH as thiazide diuretics [72]. Furthermore, an in vitro study demonstrates that amiloride downregulates RANKL-induced expression of NFATc1, a regulator of osteoclastogenesis, and attenuates RANKL-induced expression of osteoclastic marker genes [73].

Beta-Blockers

Although beta-blockers have been used for long periods of time to control cardiovascular disease and hypertension, the evidence for their benefit in the osteoporotic community is mixed. Some studies have demonstrated that B1 agents, such as atenolol and nebivolol, increase bone density compared with control patients and may be more beneficial for anti-hypertensive treatment in men older than 55 with osteoporosis than CCBs [74,75]. Although beta-blockers are not the first line of therapy for hypertension, they could be a potential treatment for patients with osteoporosis and hypertension. 

Osteoporosis Medications

Along with antihypertensives, there are a few osteoporosis therapies that have also been researched for their effects on the cardiovascular system. Overall, many have a neutral or a limited impact on blood pressure. For example, raloxifene showed promise in animal models, but in human clinical trials, it failed to affect hypertension. However, it was noted that it may indirectly influence vascular risk by reducing low-density lipoprotein (LDL) and improving the function of nitric oxide on the endothelium [76,77]. Sclerostin inhibitors like romosozumab have increased benefits in helping reduce the risk of fractures; however, phase 3 trials and meta-analyses have demonstrated an increased risk of myocardial infarction and stroke, although the mechanism is poorly understood [78,79]. 

Discussion

This review highlights several key mechanisms that link hypertension and osteoporosis with the RAAS and oxidative stress. Evidence from human observational cohorts and animal studies has demonstrated how RAAS activation via angiotensin II stimulates osteoclastogenesis with the help of RANKL and NFKB signaling, along with the inhibition of osteoclast activity [44,80]. Oxidative stress has also demonstrated increased endothelial dysfunction, sympathetic activation, and increased bone resorption via NFKB and MAPK pathways [17,81]. Hormonal factors such as estrogen deficiency and calcium imbalance further amplify these processes in postmenopausal and elderly populations [18,82]. Together, these findings underscore an overlap involving RAAS dysregulation and redox imbalance, which demonstrate combined vascular and skeletal effects.

The overlap of these mechanisms has important therapeutic applications that can address osteoporosis and hypertension simultaneously, particularly focusing on the RASS system and oxidative stress. The mechanistic overlap that has been previously mentioned also has helped demonstrate that the most effective therapeutic intervention for both osteoporosis and hypertension would be ACEis or ARBs due to their ability to lower blood pressure and mitigate bone loss [44]. Along with their ability to lower blood pressure, they have also been shown to decrease fracture risk, something common in the geriatric population [46,83].

Although ACEis and ARBs have demonstrated the most evidence of a positive impact on bone, other antihypertensive medications have also demonstrated promising results. For example, thiazide diuretics increase calcium reabsorption and increase the formation of bone retention [47,48]. Calcium channel blockers have been shown to control blood pressure and decrease osteoclast activity [83]. These four examples and more observed in Table 2 of antihypertensives demonstrate the high impact and implications between osteoporosis and hypertension, which could further aid in the management of polypharmacy in patients with other comorbidities. It is important to note that the protective effects of the antihypertensives have been mostly observed in observational cohort studies and preclinical research; more randomized controlled trials would be beneficial.

**Table 2:** Effects of anti-hypertensive medications on bone health

Medication Class	Primary Mechanism of Action	Impact on Bone Health	Mechanisms Related to Bone Health
Thiazide diuretics	Decreases the reabsorption of sodium in the distal convoluted tubule, increasing natriuresis	Positive impact	Decrease calcium excretion and stimulate bone formation via decreased stimulation of parathyroid hormone (PTH)
Calcium channel blockers	Non-dihydropyridines (NDHP): Inhibit L-type calcium channels in the heart predominantly, with some effects on vascular smooth muscle. Dihydropyridines (DHP): Inhibit L-type calcium channels in the vascular smooth muscle predominantly, with decreased effects on the heart	Positive impact	Cilnidipine (DHP): Decreased osteoclast activity. Nifedipine (DHP): Induction of peroxisome-proliferator-activated receptor γ coactivator 1-α (PGC-1α) Benidipine (DHP): Inhibition of preosteoclast activity
Angiotensin-converting enzyme inhibitors (ACEi)	Inhibition of ACE reduces vasoconstriction and aldosterone secretion	Positive impact	Decreased bone resorption and increased bone mineral density
Angiotensin II receptor blockers (ARBs)	Inhibition of angiotensin II type 1 and 2 receptors	Positive impact	Increased osteoprotegerin activity and NF-κB, and decreased osteoclast markers like TRAP and cathepsin K
Potassium-sparing diuretics	Inhibit ENaC in the collecting duct, reducing sodium reabsorption while sparing potassium	Positive impact	Decreased calcium excretion, PTH activity, and RANK-L activity
Hydralazine	Relaxation of arterial smooth muscle via inhibition of IP3	Neutral	The osteoclastogenesis mechanism involves IP3, so there could be an overlap. However, more investigation is necessary
Clonidine	An alpha 2 agonist in the nucleus tractus solitarius and rostral ventrolateral medulla leading to decreased sympathetic outflow	Neutral impact	Conflicting research demonstrates positive and negative impacts on bone health
Minoxidil	Opens ATP-sensitive potassium channels, decreasing peripheral resistance	Positive impact	Shared pathway with Wnt/B-catenin which modulates RANKL and osteoprotegerin
Beta-blockers	Inhibition of B receptors found in the heart, vessels, lungs, and kidneys. Beta 1: decreased heart rate, contractility, cardiac output, and renin release. Beta 2: bronchoconstriction and vasoconstriction	Positive impact	Increased bone mineral density was observed with atenolol and nebivolol
Alpha-blockers	Inhibition of the alpha 1 receptor in blood vessels decreases norepinephrine release	Neutral	No correlation has been observed

Although this review provides an overview of the mechanisms between osteoporosis, hypertension, and therapeutic interventions, some limitations should be acknowledged as well. One includes how much of the evidence available is from observational studies, while randomized trials are limited. Along with this, a variability in populations, different classes of medication studies, and loss to follow-up are also big factors that can change the outcomes. We believe future research should continue to clarify relationships between osteoporosis and hypertension, especially within the RAAS pathway and oxidative stress in human studies. Increased clinical trial numbers can help provide integrated treatment strategies for patients with both comorbid conditions.

## Conclusions

Emerging evidence indicates that the RAAS and oxidative stress are the most strongly supported mechanisms linking hypertension and osteoporosis, with hormonal imbalance and nutritional factors contributing secondarily. Understanding these overlapping pathways highlights opportunities for dual-benefit therapies, such as antihypertensive agents like thiazides and RAAS blockers that also promote bone preservation. Recognizing this connection could help make more integrated screening strategies, such as looking at bone density in hypertensive patients and monitoring blood pressure in patients with osteoporosis. Future research should aim to focus on longitudinal and interventional studies to clarify the accurate correlation and possibly develop pharmacological approaches to target both comorbidities.
